# MiR-25 enhances autophagy and promotes sorafenib resistance of hepatocellular carcinoma via targeting FBXW7

**DOI:** 10.7150/ijms.67352

**Published:** 2022-01-01

**Authors:** Xiaoning Feng, Bei Zou, Tianhao Nan, Xiaoxiao Zheng, Li Zheng, Jiahua Lan, Wei Chen, Jun Yu

**Affiliations:** 1 Department of Hepatobiliary and Pancreatic Surgery, The First Affiliated Hospital, Zhejiang University School of Medicine, Hangzhou, 310003, China.; 2 College of Life Sciences, Zhejiang Chinese Medical University, Hangzhou, 310053, China.; 3 Cancer Institute of Integrated Traditional Chinese and Western Medicine, Key Laboratory of Cancer Prevention and Therapy Combining Traditional Chinese and Western Medicine of Zhejiang Province, Zhejiang Academy of Traditional Chinese Medicine, Tongde Hospital of Zhejiang Province, Hangzhou, 310012, Zhejiang, China.

**Keywords:** Hepatocellular carcinoma (HCC), MicroRNAs (miRNAs), FBXW7, Autophagy

## Abstract

Sorafenib resistance is a major challenge in the treatment of patients with advanced hepatocellular carcinoma (HCC). MicroRNAs (miRNAs) are a large family of non-coding RNA molecules, which is an important mechanism of drug resistance. We previously found that knockdown of miR-25 increased the sensitivity of TRAIL-induced apoptosis in liver cancer stem cells. We aimed to study the effects of miR-25 on sorafenib resistance of HCC and the underlying mechanisms. In the present study, we analyzed the expression of miR-25 between HCC and normal tissues and predicted miR-25 target genes through databases. After transfecting miR-25 mimics, inhibitor or FBXW7 Plasmid, CCK-8 and flow cytometry assay was performed to determine the sorafenib resistance. We performed LC3-dual-fluorescence assay and Western blotting to detect the autophagy levels. The expression of miR-25 was upregulated in human HCC tissues and was associated with tumor pathological grade, clinic staging, and lymphatic metastasis. MiR-25 enhanced sorafenib resistance of HCC cells and autophagy. FBXW7 is the direct target of miR-25. Overexpression of FBXW7 could reverse the increase of sorafenib resistance caused by miR-25 mimics. Our results suggested that miR-25 increased the sorafenib resistance of HCC via inducing autophagy. In addition, miR-25 decreases the expression of FBXW7 protein to regulate autophagy. Therefore, miR-25 may represent a novel therapeutic target for the treatment of HCC.

## Introduction

Hepatocellular carcinoma is a prevalent malignancy and the third cause of cancer-related deaths 1. The majority of HCC patients usually develop advanced-stage HCC due to underlying chronic liver disease, late diagnosis, and high frequencies of recurrence after treatment 2. Sorafenib, the multi-tyrosine kinase inhibitor, is the first-line systemic target drug that plays an important role in the management of advanced HCC 3. Sorafenib can prolong the median survival time of patients by 3-5 months, but drug resistance minimizes its therapeutic benefits 4. Therefore, it is important to explore the resistance mechanism of sorafenib and develop novel molecular targets for the treatment of HCC.

MicroRNAs (miRNAs) are a large family of non-coding RNA molecules that adjust cellular differentiation, proliferation, migration, and death by suppressing mRNAs expression or transcription 5. Previous research has established that several miRNAs could regulate the development of HCC drug resistance, and serve as original therapeutic targets for HCC 6. MiR-25 is dysregulated in several malignancies, including breast cancer, including breast cancer, human prostate cancer, cholangiocarcinoma, and HCC 7-9. miR-25 is over-expressed in triple-negative breast cancer (TNBC) and promotes TNBC cell proliferation. We previously found that miR-25 is overexpressed in liver cancer stem cells (LCSCs) and can reduce TRAIL-induced apoptosis via PTEN/PI3K/Akt/Bad signaling pathway 10. However, the role of miR-25 in the chemoresistance of HCC remains poorly understood.

Autophagy is a lysosomal degradation pathway, which degrades cytoplasmic constituents and organelles 11. Autophagy displays opposing functions at different cell types, which protects against HCC initiation, but induce growth, metastasis, and drug resistance during tumor progression 12. Recent work has established that autophagy can be induced by anticancer therapies, which creates a novel mechanism of chemoresistance in cancer 13. Studies showed that several miRNAs regulate the therapy response of cancer cells by inducing autophagy 14-16.

In the present study, we found that miR-25 was dysregulated in HCC and regulate the sorafenib sensitivity of HCC. Furthermore, we attempted to uncover the underlying mechanisms between miR-25 and sorafenib resistance.

## Materials and methods

### Patients and specimens

Human liver tissue samples were achieved from 28 HCC patients who received resection at the First Affiliated Hospital of Zhejiang University School of Medicine after obtaining a written informed consent.

### Cell lines and reagents

Huh7, HepG2, SNU387, and SNU449 cells were obtained from the ATCC (Manassas, VA, USA). Huh7 and HepG2 cells were cultured in DMEM medium containing 10% fetal bovine serum (Gibco, USA). SNU387 and SNU449 cells were cultured in RPMI 1640 medium containing 10% fetal bovine serum. All cell lines were cultured at 37 °C in a humidified atmosphere containing 5% CO2.

### Quantitative real-time polymerase chain reaction (qRT-PCR)

Total RNA was isolated using TRIzol (Takara, Japan) Reagent ®. Reverse transcription of miRNA was conducted using a Mir-X miRNA qRT-PCR TB Green® Kit (Takara, Japan,) according to the manufacturer's instructions. Primers for miR-25 were designed and purchased from Takara. qRT-PCR was performed on an ABI Prism 7900HT Real-Time System (Applied Biosystems Inc; Shanghai, China). Results were analyzed using the 2-ΔΔCt method. The level of miR-25 expression was normalized to U6 RNA. The detailed sequences are listed as follows:

FBWX7-homo-F

Forward primer: 5'-CACTCAAAGTGTGGAATGCAGAGAC-3'

Reverse primer: 5'-GCATCTCGAGAACCGCTAACAA-3'

miR-25-3p sequence: 5'-CAUUGCACUUGUCUCGGUCUGA-3'

### Cell viability assay

Normal or transfected HCC cells were seeded into 96-well plates and incubated overnight. After treatment with different concentrations of sorafenib for 48h, cell viability was assessed using a cell counting kit-8 assay (CCK-8; Dojindo; Kumamoto, Japan) according to the manufacturer's protocol. The absorbance in each well was measured at 570 nm using a microplate reader (Dynex, Chantilly, VA, USA).

### siRNA and transfection

The miR-25 mimics and their negative control (NC), and miR-25 inhibitor and their inhibitor NC were purchased from GenePharma (Shanghai, China). HCC Cells were transfected for 6h with mimics, inhibitor using Lipofectamine 2000 (Invitrogen, USA) according to the manufacturer's instructions. miR-25 mimic and miR-25 inhibitor were used to overexpress or inhibit miR-25 expression in HCC cells after being verified by real-time PCR analyses. The detailed sequences are listed as follows:

siFBXW7-Homo-1285:

Forward primer: 5'-GCUGAAAGGACAUGAUGAUTT-3'

Reverse primer: 5'-AUCAUCAUGUCCUUUCAGCTT-3'

siFBXW7-Homo-1776

Forward primer: 5'-GGCAUACUAAUAGAGUCUATT-3'

Reverse primer: 5'-UAGACUCUAUUAGUAUGCCTT-3'

siFBXW7-Homo-2118

Forward primer: 5'-CGGGUGAAUUUAUUCGAAATT-3'

Reverse primer: 5'-UUUCGAAUAAAUUCACCCGTT-3'

hsa-miR-25 mimics

Forward primer: 5'-CAUUGCACUUGUCUCGGUCUGA-3'

Reverse primer: 5'-AGACCGAGACAAGUGCAAUGUU-3'

hsa-miR-25 inhibitor 5'-UCAGACCGAGACAAGUGCAAUG-3'

### Flow cytometry assay

Normal or transfected HCC Cells were treated with sorafenib (IC50) for 48h. The cells were stained with the Annexin-V and PI according to the manufacturer's protocol (Dojindo; Kumamoto, Japan). The rate of apoptosis was detected on the flow cytometry according to the manufacturer's guidance.

### LC3-dual-fluorescence assay

Huh7 and SNU387 cells stably transfected with RFP-GFP-LC3 adenovirus were subjected to treatment for 48 h. the cells were fixed with 4% paraformaldehyde (Sigma, USA) and photographed using a laser confocal fluorescence microscope. Cells were detected by the expression of green (GFP) or red (RFP) fluorescence. Autophagosomes were characterized by yellow puncta and autolysosomes based on only red puncta in the merged images. Autophagic flux was monitored by an increased percentage of only red puncta in the merged images.

### Western blot assay

Protein was extracted from HCC cells, separated on 10% SDS PAGE gel and then transferred to polyvinylidene difluoride (PVDF) membranes (Millipore, Bedford, MA). The transferred membranes were blocked using 5% non-fat dry milk in TBST for 2 h and incubated with primary antibodies overnight at 4 °C. Then, the membranes were incubated for 2 h in secondary antibodies at room temperature. The primary antibodies used in this study were as follows: anti-P62, anti-LC3B, anti-FBXW7 (Cell Signaling Technology, USA). GAPDH was used as an internal control. Using a detection system of enhanced chemiluminescence (ECL) to visualize proteins.

### UALCAN database analysis

The UALCAN database (http://ualcan.path.uab.edu) is a web resource for analyzing cancer data. We used UALCAN to evaluate miR-25 expression in HCC based on tumor grade, individual cancer stage, nodal metastasis status or sample types. The miRNA was set as “miR-25”, the cancer was set as “liver hepatocellular carcinoma”, and the analysis method was set as “expression” analysis. Through setting analysis method as the “Survival” module, we obtained the effect of miR-25 expression level on LIHC patient survival.

### Prediction of miR-25 target gene

The miR-25 target gene was predicted by using three online tools, which were miRTarBase (https://mirtarbase.cuhk.edu.cn/), TargetScan (http://www.targetscan.org/) and miRwalk (http://mirwalk.umm.uni-heidelberg.de/). We obtained the common predicted target genes from the three databases by using Venn diagram. Venn diagram was drawn by R software.

### Functional Enrichment Analysis

Functional enrichment analyses of the common predicted target genes from the three databases were performed through Metascape (http://metascape.org/gp). The thresholds were set as follows: the P-value cut off was 0.01, the number of min overlap was 3, and the min enrichment was 1.5. The enriched terms with the best p-values from each of the 20 clusters were selected, with the constraint that there are no more than 15 terms per cluster and no more than 250 terms in total. The selected terms were rendered as a network plot using Cytoscape (https://cytoscape.org/). The similarity of terms connected by edges is more than 0.3.

### Statistical analysis

Every experiment was performed at least three times. All data were presented as the mean± standard deviation (SD), and were analyzed using SPSS 25.0. The data were analyzed using a two-tailed Student's t-test for average differences. Statistical significance was defined as a p-value <0.05.

## Results

### MiR-25 is overexpressed in HCC tissues

**Figure 1:**
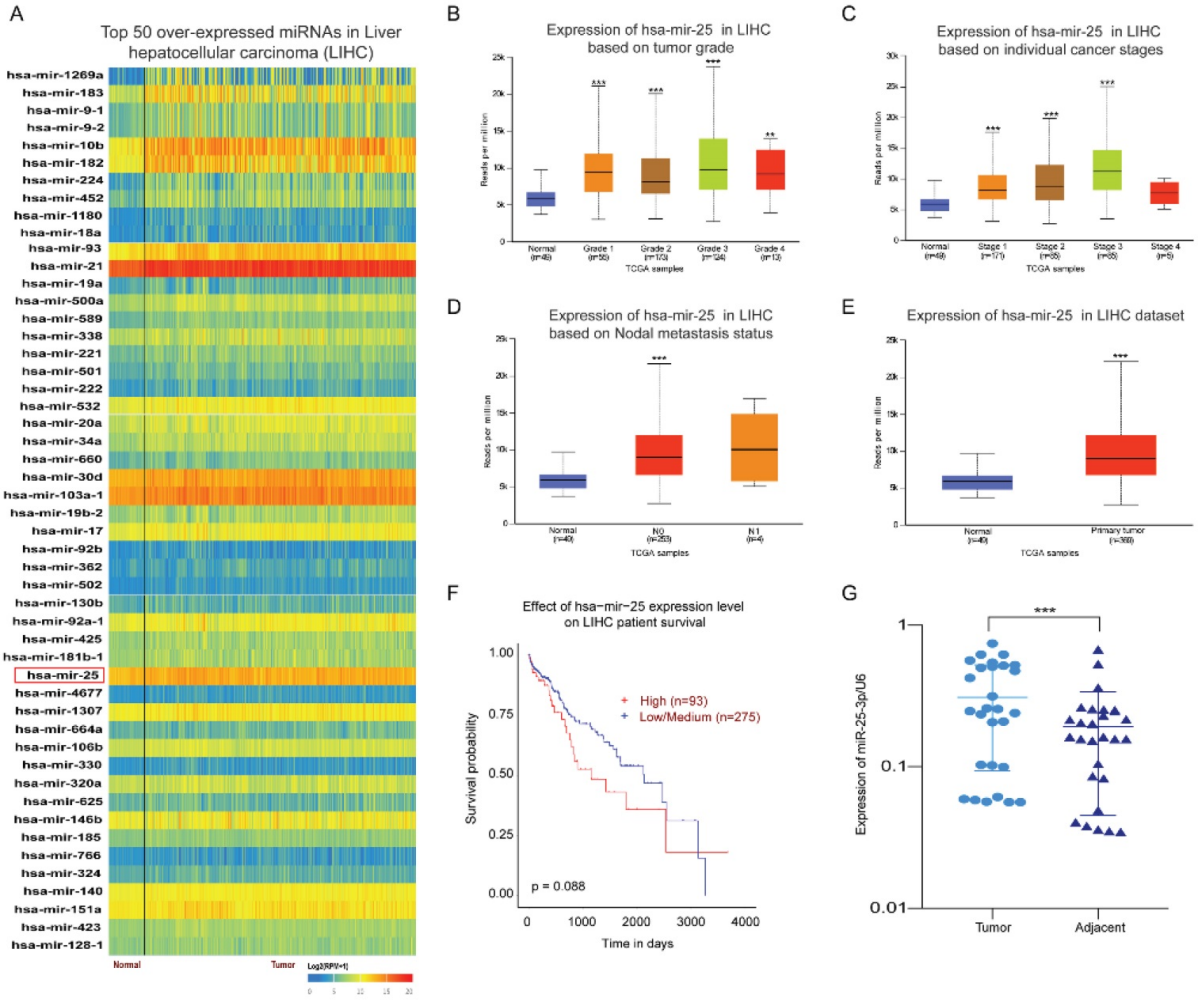
**miR-25 is overexpressed in HCC**. (A) Top 50 over-expressed miRNAs in HCC. (B-D) miR-25 expression levels in different tumor pathological staging, pathological staging, and nodal metastasis status were analyzed through bioinformatics. E miR-25 expression levels between HCC tissues and adjacent tissues were analyzed through bioinformatics. F Effect of miR-25 expression level on HCC patient survival. G miR-25 expression levels between HCC tissues and adjacent tissues were detected in clinical specimens using qRT-PCR. **p<0.01, ***p<0.001 compared with normal.

By analyzing the expression of miRNAs in HCC and normal tissues through the UALCAN database, we found the top 50 over-expressed miRNAs in HCC. The result showed that miR-25 is overexpressed in HCC tissues (Fig. 1A). Our previous article has found that miR-25 expression upregulation in LCSCs and was associated with the sensitivity of LCSCs to TRAIL 10. Therefore, we selected miR-25 for further investigation. Compared with the normal tissue, the expression of miR-25 in grades 1-4 HCC was significantly increased based on the tumor pathological staging (Fig. 1B). Moreover, the expression of miR-25 is positively correlated with the tumor clinical stages of 1-3 and the nodal metastasis status (Fig. 1C, D). miR-25 had a high expression in HCC tissues compared with adjacent tissues (Fig. 1E). The survival curve showed that HCC patients with high miR-25 expression had a worse prognosis (Fig. 1F). Furthermore, we detected the expression levels of miR-25 in HCC tissues and adjacent tissues collected from 28 HCC patients who had undergone resection and found that miR-25 was remarkably overexpressed in HCC tissues (Fig. 1G).

### MiR-25 induces resistance of HCC cells to sorafenib

**Figure 2:**
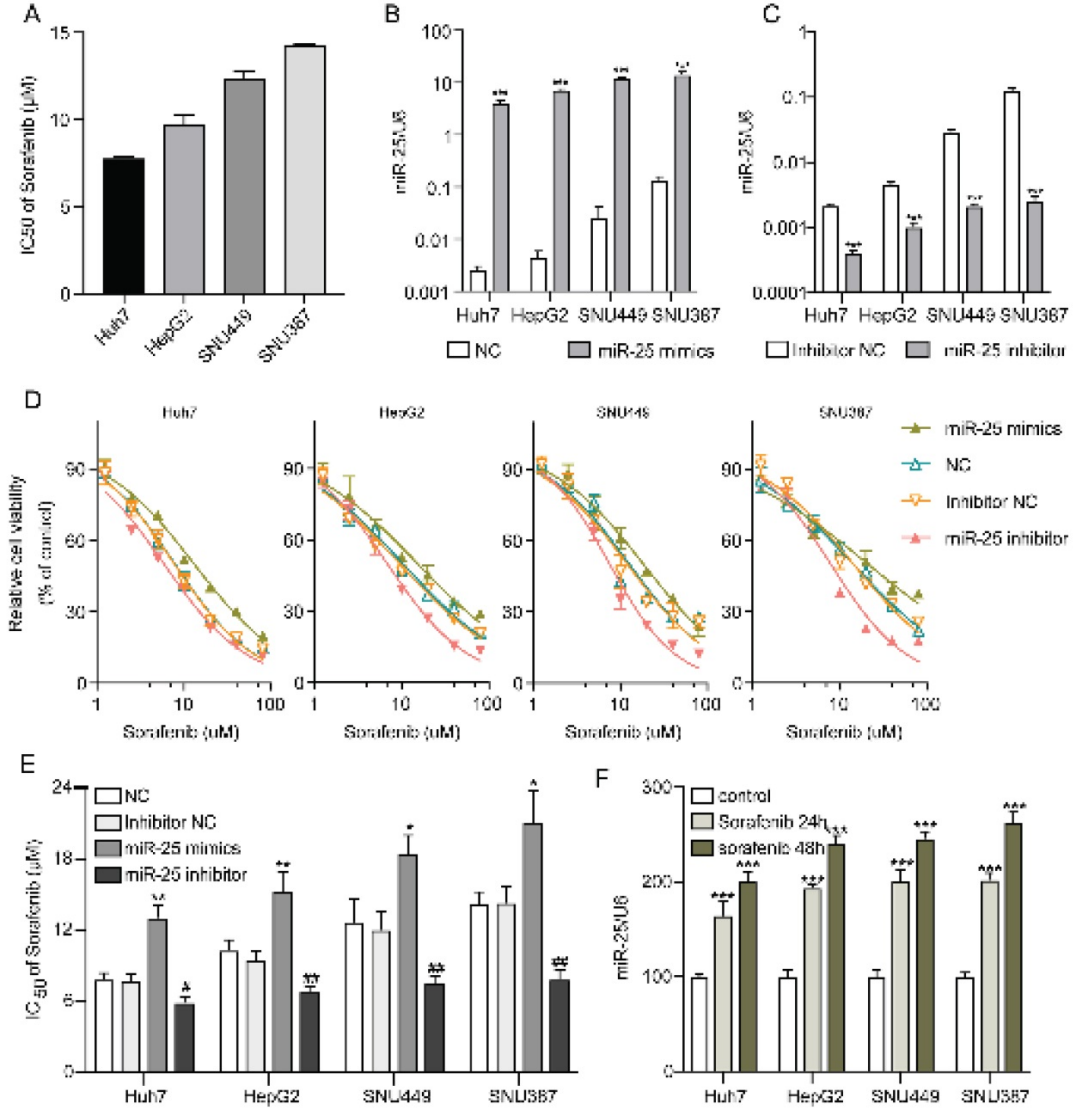
**miR-25 induces resistance of HCC cells to sorafenib.** (A) IC50s of sorafenib for different HCC cell lines. (B) miR-25 expression in the negative control and miR-25 mimic transfected HCC cell lines (***p<0.001 compared with NC). (C) miR-25 expression in the inhibitor negative control and miR-25 inhibitor transfected HCC cell lines (***p<0.001 compared with inhibitor NC). (D) Relative cell viability (mean ± SD) for miR-25 mimic or inhibitor transfected HCC cell lines with sorafenib treatment. (E) The IC50s of different HCC cell lines transfected with miR-25 mimics or inhibitor against sorafenib (**p<0.01 compared with NC; #p<0.05, ##p<0.01 compared with inhibitor NC). F the expression of miR-25 in HCC cell lines with or without sorafenib treatment (***p<0.001 compared with control).

IC50s of sorafenib for Huh7, HepG2, SNU387, and SNU449 cell lines were examined by CCK-8. The results demonstrated that Huh7 cells were the most sensitive to sorafenib, however, SNU387 cells were the most resistant (Fig. 2A). In order to investigate the role of miR-25 in the susceptibility of HCC cells to sorafenib, we altered the expression of miR-25 by transfecting miR-25 mimic, inhibitor, and corresponding negative control. The efficacy of transfection was validated (Fig. 2B, C). Then sorafenib cytotoxicity was measured following miR-25 transfection in HCC cell lines. Sorafenib cytotoxicity for the HCC cells transfected with miR-25 mimics augmented, whereas the cytotoxicity decreased in the miR-25 inhibitor transfected HCC cells (Fig. 2D). Furthermore, we measured the IC50s of different HCC cell lines transfected with miR-25 mimics or inhibitor against sorafenib. As shown in Fig.2E, the overexpression of miR-25 upregulated the sorafenib IC50s, conversely, the IC50s in HCC cells transfected with miR-25 inhibitor were lower. Moreover, the expression of miR-25 was measured in HCC cell lines with or without sorafenib treatment. The results showed that miR-25 expression was upregulated after sorafenib treatment (Fig. 2F).

### MiR-25 regulates cell apoptosis in HCC cells

**Figure 3:**
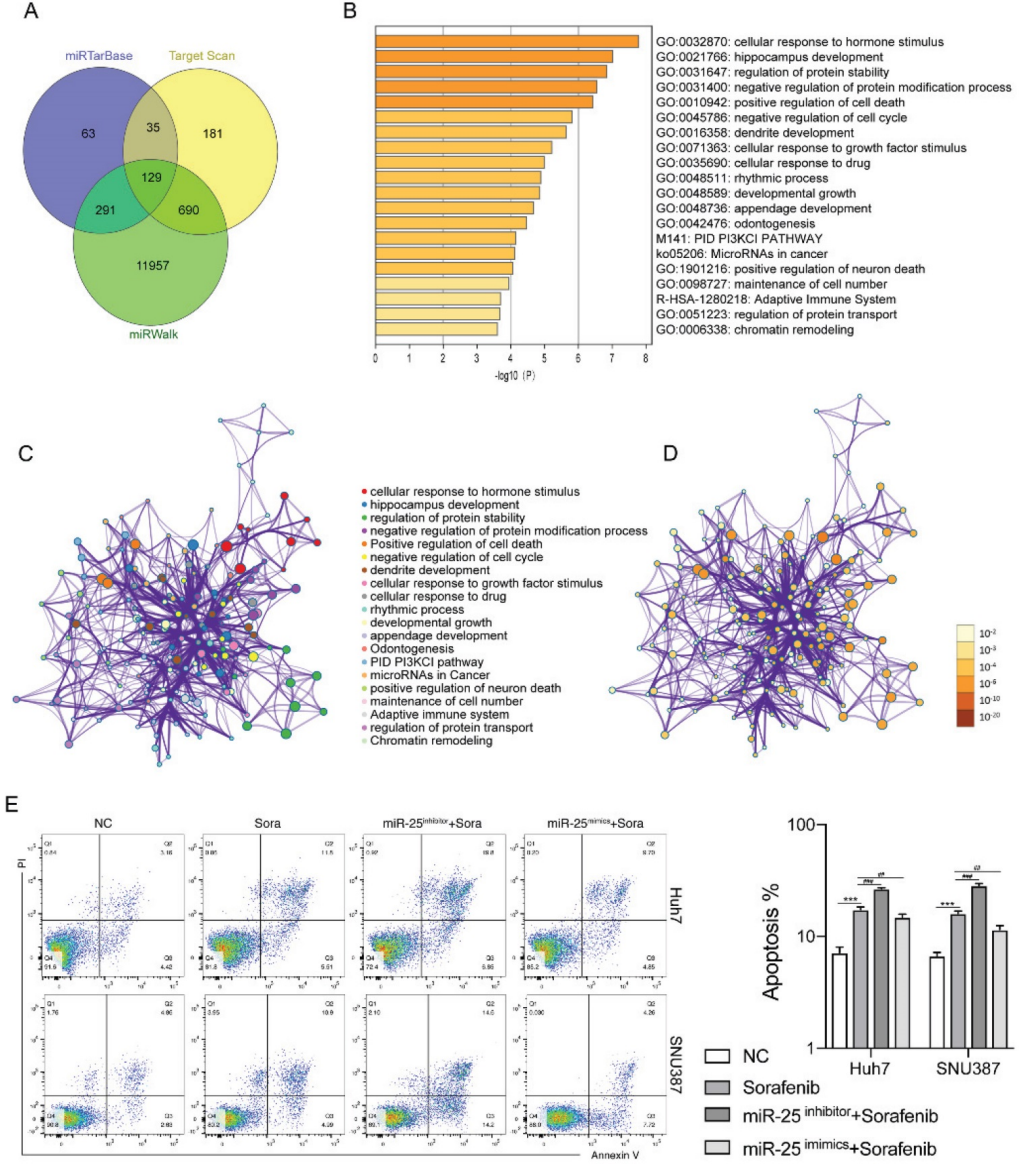
**miR-25 regulates cell apoptosis in HCC cells. (**A) The potential target genes of miR-25 using the miRTarbase, Target Scan, and miRwalk database. (B) The bar graph of functional enrichment result of the potential target genes, colored by p-values. (C, D) Network of enriched terms: (C) colored by cluster ID, where nodes that share the same cluster ID are typically close to each other; (D) colored by p-value, where terms containing more genes tend to have a more significant p-value. E The proportion of apoptosis of Huh7 and SNU387 cells transfected with miR-25 mimic, inhibitor, and corresponding negative control after treating with sorafenib (***p<0.001 compared with NC, ##p<0.01, ###p<0.001 compared with sorafenib).

We next investigated the mechanisms by which miR-25 regulates the sensitivity of HCC cells to sorafenib. Using the bioinformatics algorithm, we forecasted the potential target genes of miR-25 through the miRTarBase, Target Scan and miRwalk database. 129 common predicted target genes were obtained from the three databases (Fig. 3A). Functional enrichment analyses of the 129 common predicted target genes were performed through Metascape. It was found that the genes regulated by miR-25 were mainly related to signal pathways such as cell death and cell cycle (Fig. 3B-D). The above results suggested that miR-25 may alter the sensitivity of HCC cells to sorafenib by regulating cell apoptosis. Silence or overexpression of MIR-25 in Huh7 and SNU387 cells, and then combined with sorafenib to observe the changes in cell apoptosis. Compared to sorafenib treatment alone, the transfection of miR-25 inhibitor combined with sorafenib increased the proportion of apoptosis (Fig. 3E). Whereas, the transfection of miR-25 mimics combined with sorafenib reduced the proportion of apoptosis.

### MiR-25 regulates sorafenib-induced autophagy in HCC cells

**Figure 4:**
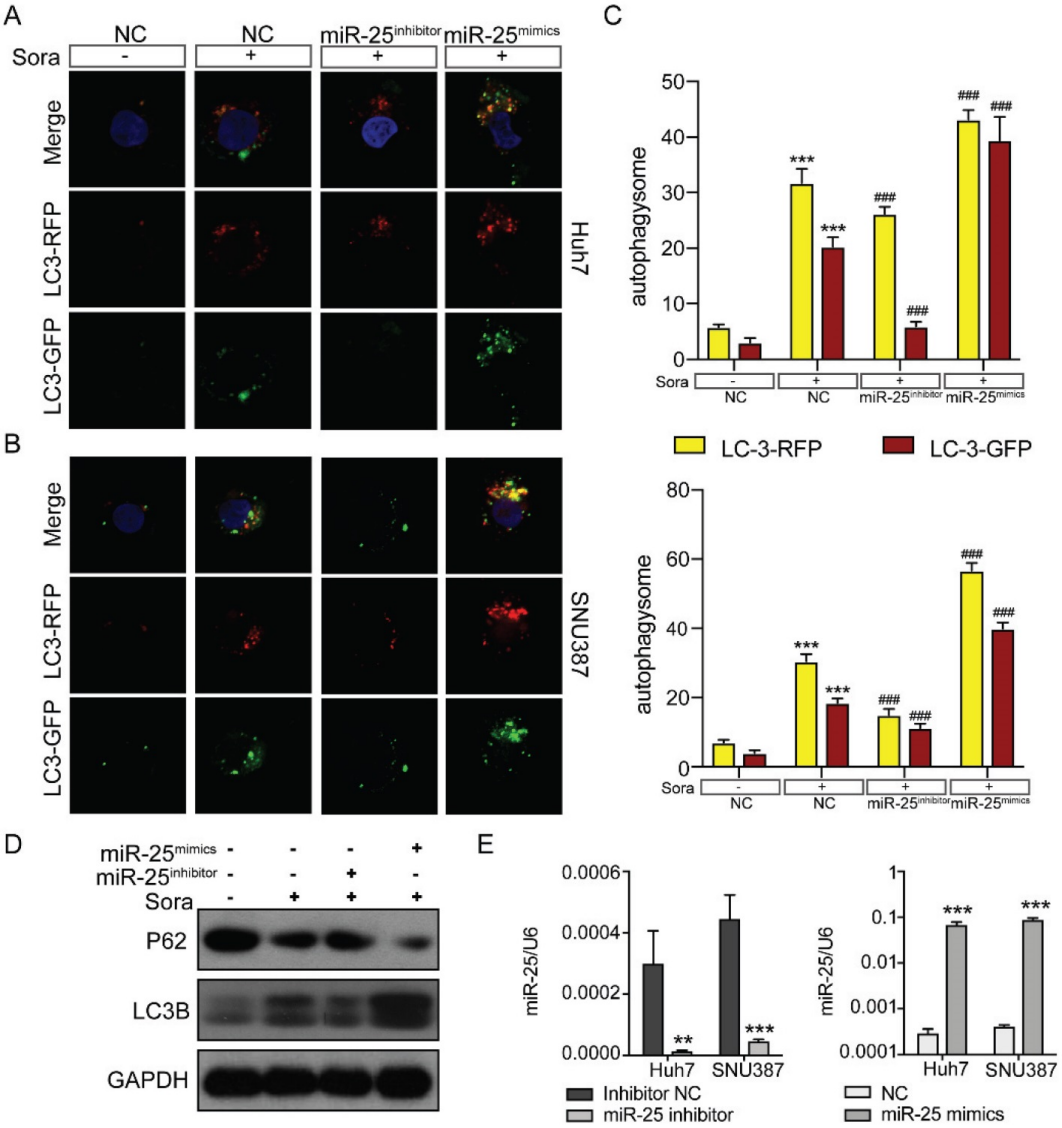
**miR-25 regulates sorafenib-induced autophagy in HCC cells.** (A-C) Autophagy flux was examined in Huh7(A) and SNU387(B) cells transfected with the miR-25 mimics, miR-25 inhibitor or NC using LC3-dual-fluorescence assay after treatment of sorafenib. The number of autophagosomes (yellow puncta) and autolysosomes (red puncta) were assessed(C). ***P<0.001 compared with NC;^ ###^P<0.001 compared with NC combined with sorafenib treatment. (D) Quantification of P62 and LC3 II protein expression in Huh7 and SNU387 cells transfected with the miR-25 mimics, miR-25 inhibitor or NC treated with sorafenib by Western blot. (E) The transfection efficiency was tested by qRT-PCR. ***p<0.001 compared with NC.

There are three classic cell death pathways: apoptosis, autophagy, and necrosis. Apoptosis and autophagy are independent and interrelated 17. As shown in Figure 3, miR-25 was involved in regulating cell apoptosis, so we then verified whether autophagy was also involved in mediating HCC cell resistance to Sorafenib. We upregulated or downregulated miR-25 expression of Huh7 and SNU387 by transfecting miR-25 mimics or inhibitor. Then we tested the autophagic flux using LC3-dual-fluorescence assay after treatment of sorafenib. The results showed that after the treatment of sorafenib, autophagy occurred in the HCC cell lines, and inhibiting the expression of miR-25 can block this effect. However, increasing miR-25 expression can further enhance autophagy compared to sorafenib treatment alone (Fig. 4A-C). Moreover, Western blots showed that the level of P62 protein and LC3 II protein was up-regulated after the treatment of sorafenib, which was reversed by miR-25 suppression (Fig. 4D). The transfection efficiency is presented in Fig. 4E.

### FBXW7 is a direct target of miR-25

**Figure 5:**
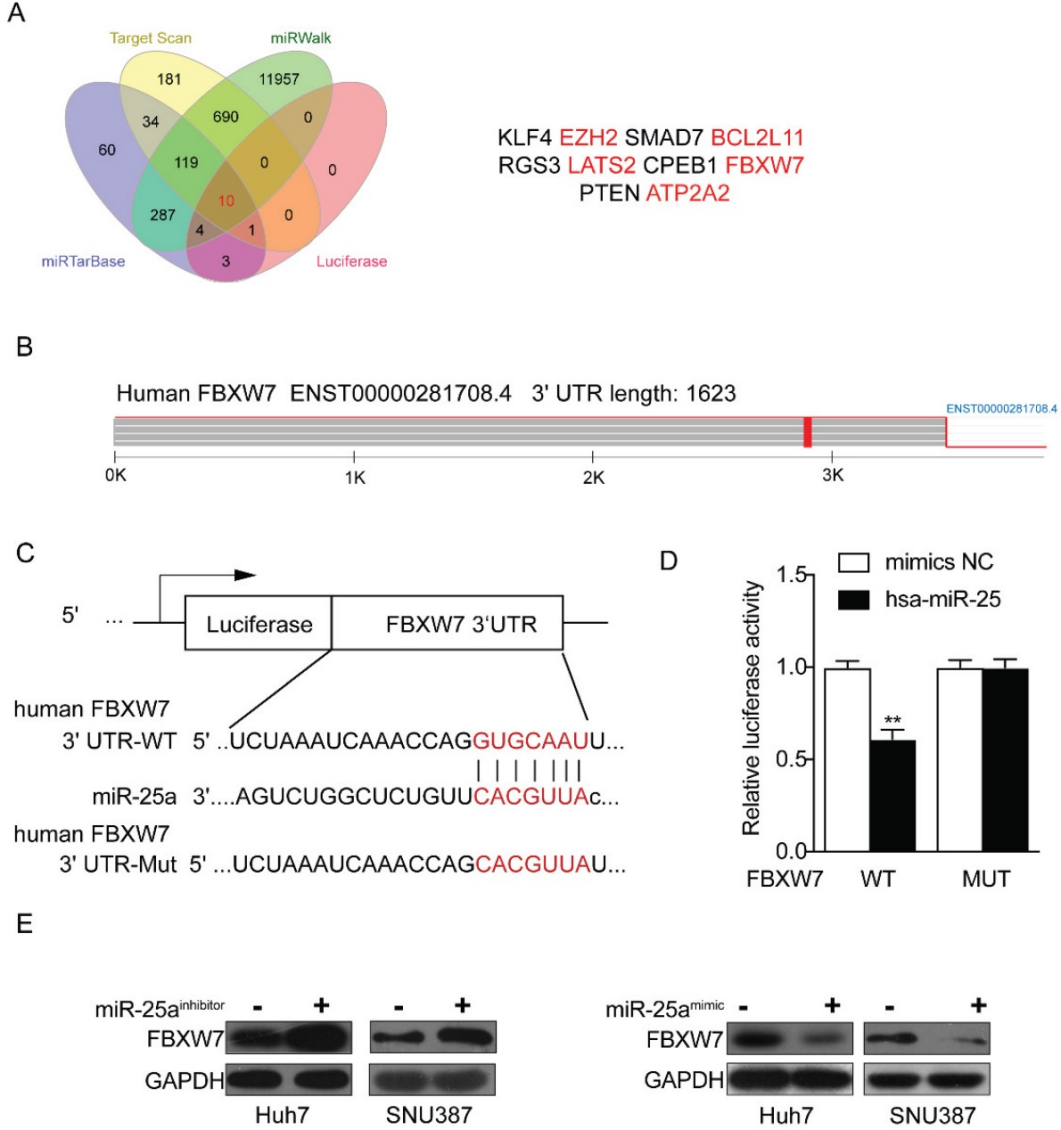
**FBXW7 is a direct target of miR-25.** (A) Ten intersections of the target genes of miR-25 were obtained from databases, including miRTarbase, Target Scan, Luciferase and miRwalk databases. (B-D) A potential target site of miR-25 on the FBXW7 3'URT was predicted by the databases. A luciferase reporter assay showed the predicted binding sequence was required for miR-25 inhibition. E Quantification of FBXW7 protein expression in Huh7 and SNU387 cells transfected with the miR-25 mimics, miR-25 inhibitor, or NC by Western.

Through the above experiments, we basically affirmed the role of miR-25 in sorafenib resistance in HCC cells. Therefore, the target genes of miR-25 needed to be explored. We sought to identify potential target genes that interact with miR-25 by studying multiple bioinformatics and databases (miRTarbase, Target Scan, Luciferase and miRwalk databases), and obtained Ten intersections (Fig. 5 A). According to the set obtained by Fig. 5 A, possible target genes were selected including FBXW7, EZH2, BCL2L 11, LATS2 and ATP2A2. Our result has verified that FBXW7 could be direct targets of miR-25 by performing a dual-luciferase reporter assay (Fig. 5B-D) as previous study 18. We transfected miR-25 inhibitor into Huh7 and SNU387 cells, which resulted in overexpression of FBXW7 protein as revealed by Western blot. Transfecting miR-25 mimic into Huh7 and SNU387 cells decreased the level of FBXW7 protein (Fig. 5E).

### MiR-25 targets FBXW7 to promote autophagy and increase sorafenib resistance

**Figure 6:**
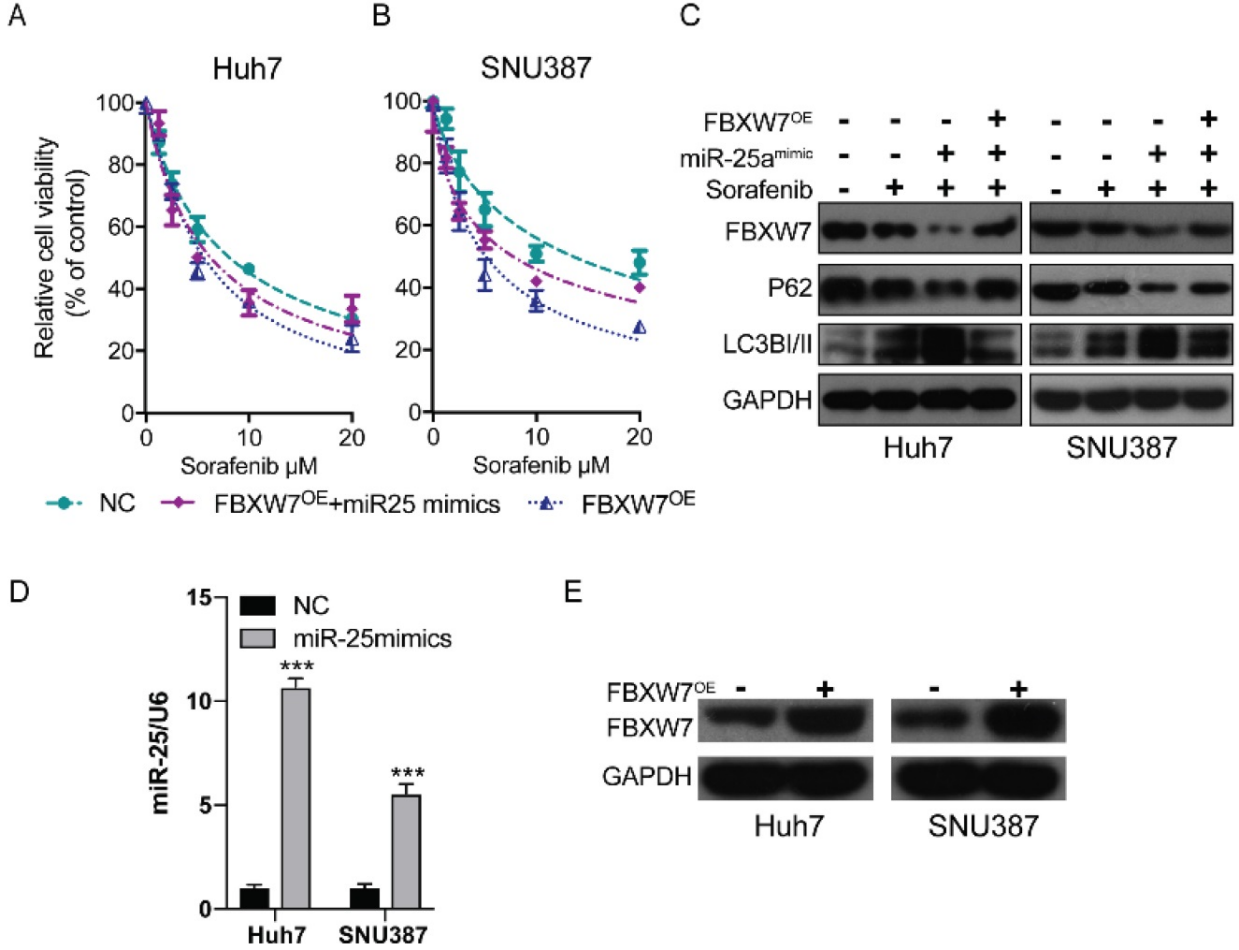
**miR-25 targets FBXW7 to promote autophagy and increase sorafenib resistance**. (A, B) Relative cell viability (mean ± SD) for HCC cell lines that overexpressed FBXW7 with or without transfecting miR-25 mimics. (C) Western blot of FBXW7, LC3-I/II, and P62 in HCC cells treated with miR-25 mimics plus transfection FBXW7 Plasmid, miR-25 mimics alone, FBXW7 Plasmid alone or NC. (D) The transfection efficiency of miR-25 mimics was tested by qRT-PCR (***p<0.001 compared with control). (E) The transfection efficiency of FBXW7 Plasmid was tested by Western blotting.

Previous research has established that the inhibition of FBXW7 expression can promote autophagy 19. We hypothesized that miR-25 may promote autophagy by mediating FBXW7 and then increase sorafenib resistance in HCC. We detected the therapeutic effect of sorafenib in HCC cells after overexpressing FBWX7. The results showed that FBWX7 overexpression increase the sorafenib sensitivity of HCC. Then the effect was assessed after overexpressing FBWX7 combined with transfecting miR-25 mimics into HCC. The results showed that overexpression of FBXW7 could reverse the increase of sorafenib resistance caused by (Fig. 2D and Fig. 6A-B). Western blotting showed that miR-25 overexpression led to the amplification of autophagy, which was reversed by the overexpression of FBXW7 (Fig. 6C). The transfection efficiency of miR-25 mimics and FBXW7 Plasmid was tested by qRT-PCR and Western blotting, respectively (Fig. 6D-E).

## Discussion

Sorafenib was approved by FDA as the first-line targeted drug for patients with advanced HCC, which can suppress cell proliferation, angiogenesis, and promote tumor cell apoptosis. Although sorafenib prolongs the median survival time of advanced HCC patients, the overall outcomes are not satisfactory with the resistance to sorafenib 3, 20. Many recent studies have shown that the expression of miRNA is related to the drug resistance of cancer cells 21, 22. miR-25 has been reported as highly expressed in human cancer, acting as an oncogenic miRNA. In triple-negative breast cancer (TNBC), miR-25 was found to promote TNBC cell proliferation and tumor growth 7. In colorectal cancer (CRC), miR-25 induces vascular leakiness and enhances CRC metastasis to the liver and lung 23. CRC patients with metastasis have a higher expression level of miR-25. miR-25 is significantly upregulated in HCC, which increases the HCC cell viability, and are related to poor survival 24. But the role of miR-25 in sorafenib resistance in HCC remains unknown. Our current study revealed that upregulating the expression of miR-25 increases the resistance of HCC to sorafenib.

Autophagy plays a complex role in tumor survival, which limits the earliest stages of tumorigenesis, but enables tumor cell survival and growth in established cancers. Autophagy can promote the established tumors' survival and growth by helping cope with intracellular and environmental stresses, such as hypoxia, nutrient shortage, or cancer therapy 25. Recent evidence has indicated that autophagy as a cellular defense mechanism is associated with the development of resistance in multiple cancers. Induction of autophagy leads to tumor chemoresistance through different mechanisms, including EGFR signaling, PI3K/AKT/mTOR pathways, and microRNA 26. Evidence has shown that MIR30A enhances chemoresistance against chronic myelogenous leukemia via promoting autophagy 27. Moreover, inhibition of autophagy enhances the anti-tumor activity of temozolomide and rapamycin on glioma stem-like cells 14. Regarding HCC, several studies have demonstrated an association between miRNAs and autophagy in the chemosensitivity of HCC 28, 29.

F-box and WD repeat domain containing 7(FBXW7), one of the ubiquitin-proteasome system proteins, is an essential tumor suppressor and is frequently deregulated in human cancer cells 30. Evidence has shown that miRNAs can bind to the 3' untranslated region (UTR) of FBXW7 and target the mRNA for degradation and prevent protein translation. MIR-223 has been shown to bind directly to the 3' UTR of FBXW7 and inhibit the level of FBXW7 protein expression 19. In HCC, miR-92a negatively regulated FBXW7 abundance, which promotes proliferation, cell cycle transition and apoptosis resistance 31. In endogenous cardiomyocyte, miR-25 is proven to target directly FBXW7 by using the luciferase reporter assay. The overexpression of miR-25 resulted in downregulation of FBXW7 expression 18. Several studies demonstrated that FBXW7 regulated the autophagy in several diseases 32, 33. Study has shown that the down-regulation of FBXW7 proteins can promote autophagy 19. However, there are no reports on the role of miR-25 and FBWX7 in the regulation of autophagy. In the present study, our results demonstrate that miR-25 can induce autophagy by targeting FBXW7 directly.

In conclusion, we showed that miR-25 induced autophagy and chemoresistance in HCC by targeting FBXW7. These results indicate that miR-25 may be a valuable target to overcome sorafenib resistance in HCC.
